# An Essay on Inflammation

**Published:** 1834-04-01

**Authors:** 


### II.—(BIS.)

**AN ESSAY ON INFLAMMATION; BEING AN INQUIRY INTO THE CAUSES, PHENOMENA, TREATMENT, AND TERMINATIONS OF THIS CONDITION, WITH A VIEW TO THE ELUCIDATION OF THE PROXIMATE CAUSE.** By *Philip Lovell Phillips*, M.D. Oxon. Octavo, pp. 153. London, 1833.

WE know Dr. Phillips to be able, ardent, and industrious. He has studied in a good school, St. George's, and what he has studied he has made his own. Yet we cannot agree with him in the theory which it is the object of his work to establish, on which he has expended infinite pains, and to which he has devoted considerable time.

That theory is the doctrine of debility of the capillaries as the proximate cause of inflammation. It comes recommended by high authority—founded on plausible experiments—strengthened by seductive and logical reasoning—and wearing that aspect of exactness and of demonstration that carries unwilling scepticism by assault.

Yet experience and observation, those sullen scorners of the blandishments of theory, suggest objections to the one before us—objections that have never been triumphantly refuted—objections that have been answered by appeals to experiments rather than to Nature.

There is much in experiments to captivate; history tells us there is even more to mislead. There is scarcely a physiological absurdity that has not been supported by an army of experiments. They are to physiological science what cases have been to medicine—teeming with fallacy and falsehood. By experiment, Majendie determined that the fifth was the nerve of smell—by experiment, he proved that an animal is under several antagonizing impulses: to go forward, backward, laterally, to revolve—by experiment, he concluded that there was a fluid in the spine and in the ventricles, which, becoming cold, produces ague. In short, there is no folly, no heresy, that cannot fall back on experimental *facts*.

We do not undervalue experiments. We esteem them most highly, but we depend upon them *only* when they chime with the great phenomena of Nature. Those phenomena are themselves a grand series of experiments, conducted in her laboratory, and open to the common observation of mankind. It is true that her processes are generally secret, often unintelligible. Yet the results are obvious, and many of the links in the chain of causation may be seen and handled.

The theory of the proximate cause of inflammation which Dr. Phillips advocates makes debility of the capillaries the efficient agent. The action of the heart and great vessels may be natural or altered, but debility of the capillaries is the circumstance that is essential. The experiments on which this hypothesis is grounded may be said to express the following conditions.

1. That, on the application of some stimulants, paleness is the first result, and redness may or may not follow.

2. That other stimulants occasion redness without preceding paleness.

3. That, in the state of inflammatory redness, the blood has sometimes flowed with diminished, sometimes with increased velocity.*

These are the main facts. We turn to the principal correlative speculations.

1. It is concluded that the action of a capillary is contraction.

2. A capillary in the condition of inflammation is dilated, and as dilatation is the converse of contraction, and contraction the measure and the consequence of action and of power, it is probably in a state of debility and atony.

This is an analytical expression of the theory of debility as the proximate cause of inflammation. Stripped of its outside ornament, it is the skeleton and frame-work of the argument. Let us see how it agrees with the natural phenomena.

In practice we find two conditions opposed to each other, yet insensibly mingling at their respective confines. These conditions are inflammation and congestion. How distinct they are in some particulars, and in most of their effects, we need not pause to explain. Yet the theory of capillary debility appears as if originally cut for congestion, and undoubtedly fits it the best. In congestion the velocity of the current of blood is confessed to be diminished—the vessels are obviously dilated—the important hypothetical conditions of inflammation are existent—yet their sum is not inflammation.

This then is one objection to the theory; it does not account for the manifest distinction between inflammation and congestion.

Inflammation is not an absolute condition, fixed in its characters, and unvarying in its features. On the one hand, it can scarcely be distinguished from congestion; the tint is dull, the circulation languid, stimulants, (which cause contraction,) remove it. On the other hand, the circumstances differ from the former; the colour of the part is of a vivid scarlet, the heat is great, the circulation hurried, stimulants aggravate the inflammatory action.

It is a second objection to the theory we are considering, that it does not explain these different kinds of inflammation.

Let us take an instance of active inflammation in an external part, and try the theory by its applicability to the several phenomena. Those phenomena are redness—increased temperature—swelling—and pain.†

---

* Some of the experimenters dislike this statement of Dr. Thomson's. They contend that when the velocity was augmented, it was not orthodox and genuine inflammation.—*Ed.*

† The succeeding arguments are contained in the number of the Medico-Chirurgical Review for October, 1832. The Reviewer may be permitted to steal from himself.

1. *Redness.* An inflamed part, if formerly white, becomes more or less red, and if previously red, it is rendered redder. This arises, of course, from the greater quantity of red blood which is in it. But we must not stop here. Venous blood and arterial differ in colour, and, for precisely the same reason, blood circulating slowly is less florid than blood circulating rapidly. In phlegmonous inflammation the colour is vivid, not only because there is much arterial blood in the part, but also because that arterial blood is frequently renewed; in other words, because it is circulated with rapidity. If an incision be made into an inflamed part, all surgeons are aware that not only does more blood escape, but that it escapes with unusual force from vessels of all calibre. It may be said that this is owing to the increased propulsive power of the heart. In inflammation sympathetically affecting the vascular system this is partly true, but in slighter inflammations, where observation can detect no alteration whatever of the general circulation, such a supposition cannot be received. It is difficult to suppose that the weakened vessels, unassisted by increase of force in the heart, would not only bleed more freely, but throw out their blood with more power.

It is a third objection to the theory that it does not inform us why blood more stagnant than natural in dilated reservoirs, should display increased redness, and flow with augmented celerity and force.

2. *Heat.* It is generally supposed that this is produced by the greater afflux of blood from the interior, because it is known that the blood of the interior is of a higher temperature than that of the surface. And, setting aside the satisfactory experiments that have been made by John Hunter and by others, this view of the cause of increased heat, is corroborated by the fact, that the cheek, when suffused with a passing blush, does glow under the temporary accession of blood from the internal parts. But if there were a stasis of blood in the inflamed part, the heat would not be maintained, for the blood being placed under the same circumstances as the surface of the body would lose its temperature, as *it* does. We may infer then, that not only more blood does arrive at the part, but also that it circulates through it with more than usual rapidity. If this be denied, then we cannot explain the phenomena of increased heat by the state of the local circulation. Throbbing, perceptible and painful, is an accompaniment of heat in inflammation. This is explained by the impulse of the heart being more sensibly felt in the dilated and weakened vessels. It is certainly present when the action of the heart is not manifestly augmented, and those who have suffered from whitlow know that it is not confined to the point immediately inflamed. It usually ceases when suppuration is established; yet we think it would be difficult to prove that the vessels then cease to be weak, if they were weak before, or to be dilated, if before dilated by the action of the heart. Mr. James remarks, that if the blood be pressed out of an inflamed part, it will return with great velocity when the pressure is withdrawn. In parts reddened from cold, where there is evident congestion, the contrary is the case.

We need scarcely express in other words this fourth objection.

3. *Swelling.* The considerations which present themselves on this phenomenon, do not prove or disprove the theory of inflammation at issue.

4. *Pain.* The pain attending acute inflammation is familiar; it is active, acute, energetic. It is explained by the pressure exercised on the nervous filaments by the enlarged blood-vessels. Probably this is not the whole truth. As the nervous system is the system of relation, that which informs both the animal and the organic life of impressions from without, it seems reasonable to imagine that the external irritant which has occasioned the phenomena of inflammation displayed by the vascular system, has also contributed to excite the sensibility of the nerves. However, this is a subject too extensive and also too subtle for us to enter on. It is evident that the nervous system is intimately linked with the vascular in inflammation. The pain attending the different varieties of inflammation varies in intensity and kind; it differs in acute pleurisy and in rheumatism, in erysipelas and in phlegmon. In certain kinds of inflammation which are called latent, pain is frequently absent. We have seen a dozen, aye, several dozen instances, in a large London Hospital, of death from latent pleuro-pneumonia, where there was little or no pain during life. In acute phlegmonous inflammation, not only is the pain pungent, but the ordinary sensibility of the part is excessively increased. Now we know that such an increase argues an augmentation of the powers of the nervous system. But such an augmentation is hardly consistent with debility in the vascular system, and what is applicable to the two systems considered each as a whole, seems fairly applicable to their parts. Thus, latent inflammations usually occur in persons exhausted by typhus, by the discharge from compound fractures, &c. and it may be imagined that it is this exhaustion, shared by the vascular and nervous systems, which renders latent inflammations unaccompanied by the ordinary nervous sensibility, and indomitable by the depletion which generally puts down vascular action. Yet with all these facts before us, we are asked to assent to the opinion that inflammation is in *all* cases essentially the same; that in every instance the smaller vessels are weakened and dilated, the circulation in them retarded.

We might advert to one of the foundations of the theory—the supposition that the action of the capillaries is contraction. We know very little of the capillary vessels—our notions of their action are founded, at the best, upon presumptions, possessed of a certain degree of plausibility; and a theory which is founded on a theory, is a house upon the sands.

There are circumstances accompanying growth and reparation, which deserve to be considered in examining the nature of inflammation.

Growth we suppose to be an active process, as it is most vigorous in youth and health, most feeble in debility and disease, almost annulled in age. There is a strong analogy between growth and reparation. During the reign of one the other is most powerful. Reparation is essentially effected by inflammation. Growth is not actually inflammation, because there are wanting in it some of the phenomena, and most of the consequences attending that process. But growth, although not inflammation, is a state approaching to it. The vessels in growth become enlarged, the circulation active, the nervous system excitable. The period of growth is the period for phlegmonous inflammation, and, as we said before, for reparative inflammation in its perfection. Here there is a strong analogy between the phenomena displayed by the vascular system in growth and in inflammation. Analogy is not proof, but it is not to be discarded in discussions, and it is always subsidiary to proof. In growth few will pretend to say that the vessels are weakened, for they are then most active, yet in inflammation, analogically comparable to growth, we are told that they are so.

Hitherto we have considered general growth, but the phenomena of partial growth, that of tumours for instance, should not be passed unnoticed. In it the blood-vessels become dilated, tortuous, the nervous sensibility occasionally increased. If we admit, and it cannot be denied, that there is general action of the vascular system in general growth, we may well imagine that there is local action of the same system in many instances of partial growth. But it will be found that, by a wise provision, local growth has a still greater connexion with inflammation than general growth. Thus morbid growths have a great tendency to inflame, and to be affected with the consequences of inflammation. Hence nature attempts to destroy them by an extension of the very same process which has produced them.

A glance at the final cause and at the consequences of inflammation may not be irrelevant to the point at issue.

The original final cause of all inflammation is probably reparation. The good God that created the animal, stamped upon his organization a tendency to actions and a power of originating them, adapted to the repair of a certain amount of injury. But it seems paradoxical to conceive, that what in itself must imply energy and vital power, should be effected by debility of the repairing agents.

The consequences of inflammation are, effusion of the albumen and fibrin of the blood—effusion of pus—effusion of new substances, as of lithate of soda in gout, the ivory deposit in certain cases of diseased joint, the phosphate and carbonate of lime in periostitis—ulceration—granulation and cicatrization—and finally mortification. Some of these processes may argue debility in the smaller vessels, others can hardly do so. Look, for instance, at the inflammation after fracture; see the bone-earth deposited, encased, moulded, and at length fashioned into nearly its natural form and consistence. Here is a process half growth, half inflammation; and it does seem to us that the smaller vessels have a power conferred on them which they did not previously possess, rather than that they are deprived of their usual quantum of it. We give this as an apposite example, but the reparative process is in principle the same, whether a bone have been broken or a finger cut, in whatever tissue, in short, it is exerted.

The treatment of inflammation should teach us something of its nature. It tells us this—that inflammation is not always the same, that acute inflammation is remedied by what empties and relaxes the large vessels and the small—that certain forms of chronic inflammation are best treated by what astringes and gives tone to them. Who does not know that phlegmonous inflammation is best treated by depletion, local or general, or both; and by the application of warmth and moisture, agents especially calculated to relax? We think this instance sufficient to upset the doctrine which makes inflammation consist in debility of vessels; at least we never heard, nor can our own poor ingenuity devise a satisfactory explanation on such principles. That inflammation is also benefited by astringents we admit; but it is not that of the most acute kind: and in discussing the proximate cause of inflammation, we are taking phlegmonous inflammation as the standard.

Dr. Phillips may complain that we have offered no analytical account of his work. We recommend all who wish to see the theory to which we have now so frequently alluded stated explicitly, and supported ably, to peruse the work itself. It does credit to the research and the judgment of its author.

---

